# A Proposal for Modeling Real Hardware, Weather and Marine Conditions for Underwater Sensor Networks

**DOI:** 10.3390/s130607454

**Published:** 2013-06-07

**Authors:** Salvador Climent, Juan Vicente Capella, Sara Blanc, Angel Perles, Juan José Serrano

**Affiliations:** Institut ITACA, Universitat Politècnica de València, Edifici 8G, 46022, València, Spain; E-Mails: jcapella@disca.upv.es (J.V.C.); sablacla@disca.upv.es (S.B.); aperles@disca.upv.es (A.P.); jserrano@itaca.upv.es (J.J.S.)

**Keywords:** underwater wireless sensor networks, simulation platform, ns-3, modeling real hardware

## Abstract

Network simulators are useful for researching protocol performance, appraising new hardware capabilities and evaluating real application scenarios. However, these tasks can only be achieved when using accurate models and real parameters that enable the extraction of trustworthy results and conclusions. This paper presents an underwater wireless sensor network ecosystem for the ns-3 simulator. This ecosystem is composed of a new energy-harvesting model and a low-cost, low-power underwater wake-up modem model that, alongside existing models, enables the performance of accurate simulations by providing real weather and marine conditions from the location where the real application is to be deployed.

## 1. Introduction

Researchers are becoming increasingly interested in underwater wireless sensor networks (UWSN). However, these networks, although sharing the same goals as terrestrial wireless sensor networks, such as network alive time or communication efficiency, present challenging new problems. Underwater communications cannot use radio frequency (RF) signals, since they have an enormous attenuation in the subaquatic medium. Therefore, acoustic signals are used underwater, which present different signal attenuations, depending on distance and frequency. In addition, signal spread is proportional to distance, due to the expansion of the wave-fronts (which may be cylindrical or spherical). Another problem in underwater communication is signal propagation, which is around 1,500 m/s and five orders of magnitude lower than in RF. In radio-frequency networks, this delay is negligible, but in underwater acoustic networks, it is significant.

Energy consumption and management is another challenge. Energy consumption is an important problem in sensor networks, as the energy available determines the main operation parameters, such as the sampling frequency, maximum distance between nodes, and so on. Moreover, given the higher energy consumption of underwater nodes, due to the transmission medium, and the difficulty of performing maintenance tasks, such as battery changes, this problem is even more challenging.

Until a satisfactory reduction of communication costs is achieved, the best option to compensate for the high energy requirements may be the use of energy harvesting mechanisms that can obtain energy from the environment to extend system operation. The flow of energy produced must fulfill the high demand of communication costs. Therefore, the energetic balance should be carefully examined and dealt with for each application.

Given these changes in the communication medium, hardware must be re-designed and new communication protocols proposed. However, several technological challenges should still be investigated for the extensive use of underwater wireless sensor networks.

All the previous advances were directed towards the construction of unattended underwater wireless sensor networks, which are of vital importance in difficult access zones, such as offshore underwater installations. New tools and methodologies are needed to tune and improve underwater installations. These new tools and methodologies need to include energy-harvesting and wake-up systems in order to improve the alive time of sensor networks. In addition, these new tools and methodologies must take into account real hardware parameters and the environmental conditions where the system is to be deployed, such as the weather or bathymetry information from the sea bottom.

In this paper, a complete UWSN ecosystem for the ns-3 simulator is presented. By extending the currently available energy and underwater frameworks of the ns-3 simulator, we are able to accurately simulate underwater applications before their development. The simulations are fed with real data (such as weather conditions, sound speed profile or bathymetry) from the location where the system will be deployed. The extensions made to the ns-3 simulator include two models based on the implemented hardware: a model of an energy-harvesting module and a model of an underwater low-cost, low-power wake-up modem.

**Figure 1.**
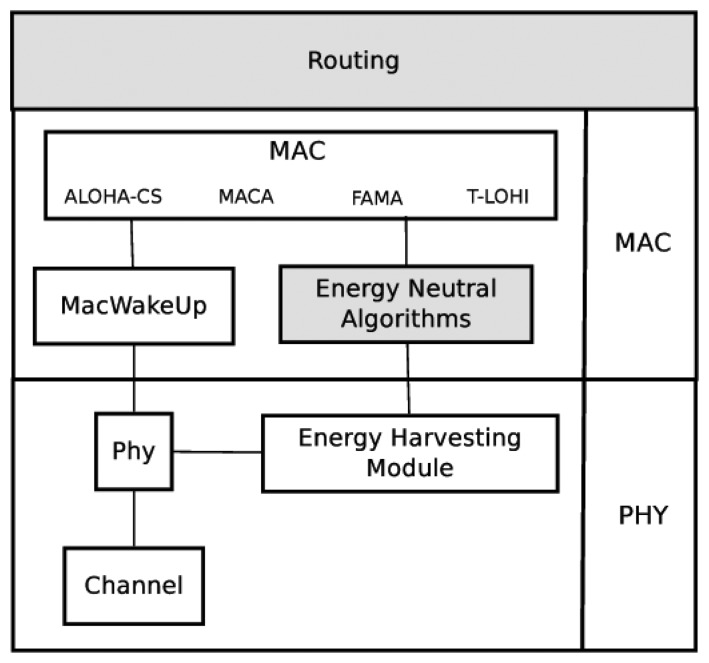
UWSN simulation ecosystem.

In Figure 1, a diagram of this ecosystem with energy-harvesting capabilities and a model of a low-cost, low-power modem is shown. The energy-harvesting module is included in the physical layer, since it is a energy-harvesting hardware model. The wake-up underwater modem implementation is divided between the physical and MAC (Medium Access Control) layers. To test this wake-up modem, different MAC protocols where implemented and adapted to use the wake-up capabilities. The gray highlighted modules are left for future work and include energy-neutral operation algorithms and routing algorithms.

The rest of the paper is organized as follows: Section 2 briefly discusses the main modeling tools for underwater networks. Section 3 introduces the ecosystems of new and already implemented models used to accurately simulate underwater sensor network installations. Section 4 shows how this ecosystem can be used in the simulation of underwater networks. Finally, in Section 5, conclusions and future work are presented.

## 2. Related Work

There are several simulators that model the underwater environment and enable the simulation and study of underwater sensor networks. Aqua-Sim [1] is a simulation package for the ns-2. This package is configured alongside the CMU wireless package for the ns-2 and has various MAC and routing protocols designed for UWSN that are ready for use.

Another simulation package for the ns-2 that enables the simulation of underwater sensor networks is ns-miracle [2]. This package also includes several protocols designed for UWSN, and when used alongside the WOSS project, can accurately model channel characteristics by including bathymetry data, salinity and sound speed profile.

In this work, the ns-3 simulator is used. The ns-3 is a network simulator written from scratch that enables the easy extension and development of new models for underwater simulation. It is designed to overcome the main drawbacks of the ns-2 simulator—such as the use of split objects (OTcl and C++), which do not scale well and complicate the debugging process. Moreover, ns-3 includes flexible tracing and logging systems, libraries to facilitate memory management, *etc.*

The ns-3 simulator includes an underwater framework that accurately models channel characteristics using, in the same way as the ns-miracle, the bellhop propagation model and environmental data, such as bathymetry or sound speed profiles. Similarly to the previously introduced simulators, the ns-3 includes a model of an underwater modem. To the best of our knowledge, there is no available underwater wake-up modem model for any of these simulators.

The ns-3 also includes an easily extensible energy framework that models various batteries and supports a range of devices; however, no model of a solar energy-harvesting module is included. Moreover, to the best of our knowledge, the currently available simulators do not include an energy-harvesting module, except for theoretical approaches [3]. For example, in [4], the authors use stationary energy levels for a one-hour period or, in [5], the authors attempt to adapt node duty cycles assuming static radiation values.

To accurately study the effects on energy consumption of using different protocols in an underwater network with energy-harvesting capabilities, it is desirable to be able to include real radiation values for days or months and analyze their impact.

## 3. Developed UWSN Ecosystem

By using the ns-3 simulator and its energy and underwater frameworks, we have developed an UWSN ecosystem that enables us to accurately simulate underwater applications before their actual development. Moreover, we are able to use data (such as weather conditions, sound speed profile or bathymetry) from the location where the system will be deployed. This data is extracted from publicly available databases to tailor the simulations.

In Figure 1, a diagram of this ecosystem with energy-harvesting capabilities and a model of a real low-cost, low-power modem is shown. The energy-harvesting module is included in the physical layer, since it is a real energy-harvesting hardware model. The wake-up underwater modem implementation is divided between the physical and MAC layers. To test this wake-up modem, different MAC protocols where implemented and adapted to use the wake-up capabilities. The gray highlighted modules are left for future work and include energy-neutral operation algorithms and routing algorithms.

This ecosystem is presented in detail below. The energy-harvesting model architecture is first introduced, followed by the wake-up underwater modem model.

### 3.1. Energy-Harvesting Model

The implementation of the energy-harvesting model is introduced [6] in this section. By using this model, it is possible to simulate several days, weeks, months or even years, using real solar radiation day curves observed in specific world locations. This data can be easily obtained by using public databases, such as the PANGAEA project [7].

**Figure 2.**
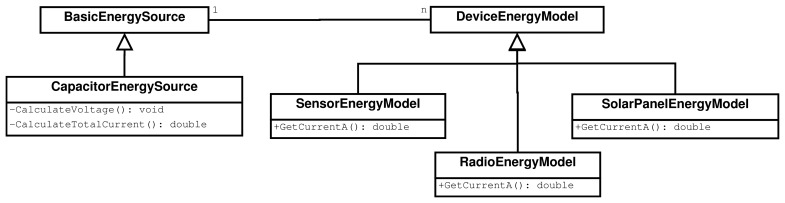
Simplified class diagram of the ns-3 energy model.

The implementation of the energy model is divided into four sub-models, each represented by one class in the ns-3 simulator. A simplified class diagram is depicted in Figure 2. The *CapacitorEnergySource* class models capacitor charge and discharge curves. The *SensorEnergyModel* class describes sensor energy consumption (temperature, humidity, *etc.*). The *RadioEnergyModel* class represents radio energy consumption at different modes, and the *SolarPanelEnergyModel* class models energy obtained from solar panels.

#### Capacitor energy source

This class manages super-capacitor charge and discharge curves using a linear model. In the *CalculateVoltage* method, the voltage that a capacitor can deliver after Δ*t* is calculated using expression (1):

(1) $$V{\text{scap}}_{t+\Delta t}=V{\text{scap}}_{t}-\frac{I{\text{scap}}_{t}}{C}\Delta t$$

where *V scap_t_* is the capacitor voltage at a certain time, *t*, and *V scap*_*t*+Δ*t*_ is the capacitor voltage after a configurable time, Δ*t*. *I scap_t_* is the difference between the current demanded by the capacitor and the current coming from the solar panel.

Component demands and current delivered by solar panels are calculated by the *CalculateTotalCurrent* method, which, at the same time, calls to the *GetCurrentA* method—which returns the current being used (or delivered, in the case of the solar model) by each component.

The *CalculateVoltage* method is always called when a mode change occurs in either the radio or sensor models. Any change causes an event to recalculate capacitor energy based on the previous state (mode) of the radio (or sensor) and the amount of time since the previous calculation was performed.

#### Radio energy model

This class models the energy consumption of a wireless radio transceiver. Four different modes have been set: reception (RX), transmission (TX), IDLE and SLEEP. Each of these modes consumes a configurable amount of current, and consumption is estimated depending on the current radio mode.

#### Sensor energy model

This class models the energy consumption of a sensor attached to the node. It has two states: enabled and disabled. In an enabled state, the sensor consumes a configurable amount of current. Meanwhile, in a disabled state, energy consumption is zero.

#### Solar panel energy model

This class models the current delivered by solar panels—which is calculated using values stored in a .csv (comma-separated values) file. Each .csv field corresponds to one-hour of mean solar radiation in *kJ*/*m*^2^. Using weather databases, such as AEMet (Spanish Meteorological Agency) or PANGAEA, we are able to easily obtain real solar radiation values.

### 3.2. Underwater Wake-Up Modem Model

An underwater modem with wake-up capabilities enables the modem to be in an ultra-low power state, while being able to recognize certain stimuli sent prior to the current data packet and, then, wake the main circuitry for reception.

The presented wake-up model is based on the ITACA modem [8] and was previously introduced in [9]. It has been designed as an energy-efficient architecture for small/medium range networks with low-power UWSN consumption restraints. Its architecture is based on a microcontroller (MCU) that only consumes 24 *mW* in reception and 3 *μW* in the sleep state. It is capable of transmitting up to 100 *m* using a frequency-shift keying (FSK) modulation at 1 *kbps*, while consuming 120 *mW*.

The acoustic wake-up signal is transmitted using an on-off keying (OOK), which is compatible (without additional hardware) with the FSK modulation used for regular transmissions. As a result, this modem also transmits the wake-up signal in the same frequency band as the regular signal.

To be able to handle acoustic wake-up signals, the ITACA modem includes an off-the-shelf commercial peripheral, the AS3933 from Austria Microsystems [10]. Since this circuit is intended to be triggered using magnetic coupled signals, a net was specifically designed to adapt the acoustic incoming signals to the RFID-based wake-up circuit. This integrated circuit with the adaptation net consumes 8.1 *μW*.

In addition to the wake-up tone capability, this modem can, with the same energy consumption, be programmed to send various wake-up patterns for selective wake-ups and only activate the receiving node.

**Figure 3.**
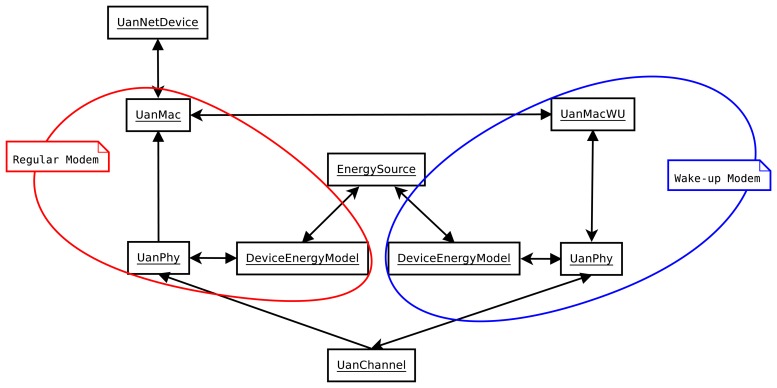
Simplified component diagram of the proposed underwater wake-up model.

Figure 3 depicts a simplified component diagram of the wake-up modem model, and further details can be found in [9]. As in the regular model, there is one UanNetDevice acting as a network interface card (NIC) card and one UanChannel to emulate the underwater channel. To model the regular modem and the wake-up system, two UanPhys and two UanMacs are introduced to model both systems.

The two UanPhy modules are equal in functionality, and the only differences are the consumption parameters (set to match the consumption parameters of the regular modem and the wake-up system). In turn, the UanMacWU module is responsible for channel assessment and sending the wake-up packet before the packet that the UanMac module sends.

The UanMac module is an abstract module. Any class implementing this module should implement the medium access algorithm. The module must encapsulate the data to be sent (namely, the data from the upper layers or the control packets, such as RTS or CTS) into an UanPacket and must ask the UanMacWU module if the channel is free. If it is free, the UanMac module sends the packet to the UanMacWU module. If it is not free, the UanMac module must decide what to do with this packet: discard it, back-off, *etc.*

**Figure 4.**
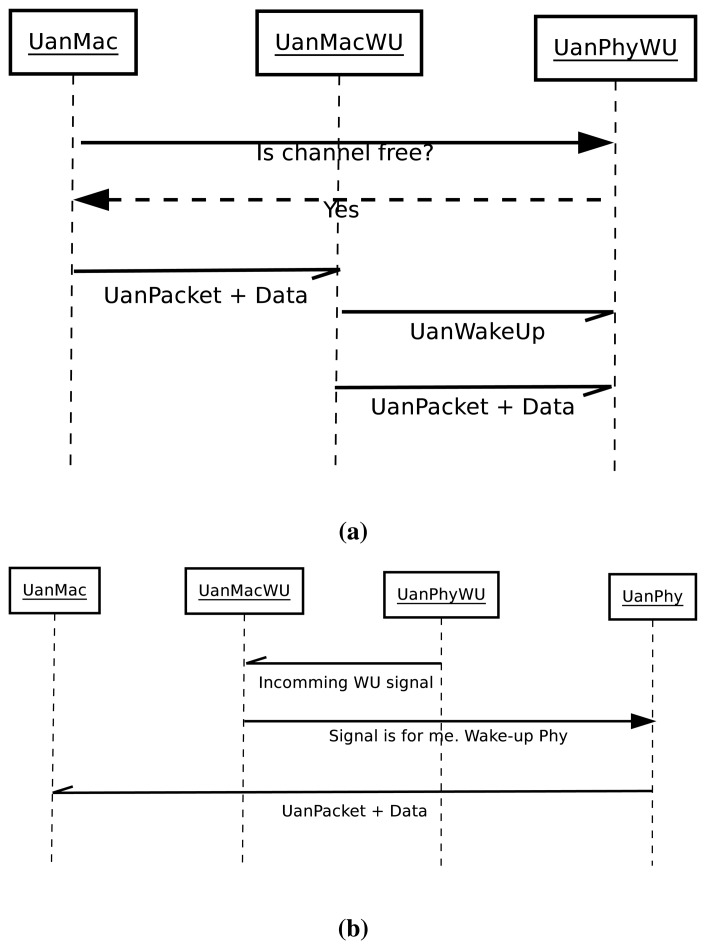
Interaction diagram between the radio model and the wake-up model. (**a**) Sending sequence; (**b**) Receiving sequence.

When a packet is received from another node, it will always be preceded by an UanPacketWU. Therefore, after reception, the UanMacWU will check if the packet destination is correct. If it is not correct, it will discard the packet. If it is correct, it will wake the UanPhy running the regular radio, so it can receive the packet. Figure 4 shows an interaction diagram between the regular radio model and the wake-up model.

In Figure 4(a), the interaction diagram for the sending sequence is depicted. It can be seen how the UanMac module asks the UanMacWU for the current channel status, and if the channel is free, it sends its UanPacket with the data from the upper layers, or its own control packets, to the UanMacWU. The UanMacWU then sends the wake-up packet, immediately followed by the UanMac packet.

The receiving sequence is shown in Figure 4(b). The UanPhyWU receives the wake-up signal from the sending node. If the signal is for this node (that is, it is a wake-up tone or a wake-up pattern that matches its own pattern), it wakes the UanPhy associated with the UanMac module, so it can receive the packet and hand it directly to the UanMac.

To demonstrate the utility of this model, a study of different MAC protocols is performed in Section 4.3 (in which the protocols have been adapted to make use of the underwater wake-up capability). Since accuracy is a major concern, simulations were carried out employing the Bellhop propagation model and extracting the SSP and bathymetry data from the location where the application will be deployed using the WOSS API [11].

## 4. Experimental

In this section, the utility of the previously presented models is shown by analyzing their usefulness in an example scenario of a real monitoring installation in an offshore fish farming facility. A brief description of the scenario is given, followed by simulations using the energy-harvesting module. Finally, an extensive study on the performance of MAC protocols using an underwater wake-up modem is shown.

### 4.1. Simulated Scenario

**Figure 5.**
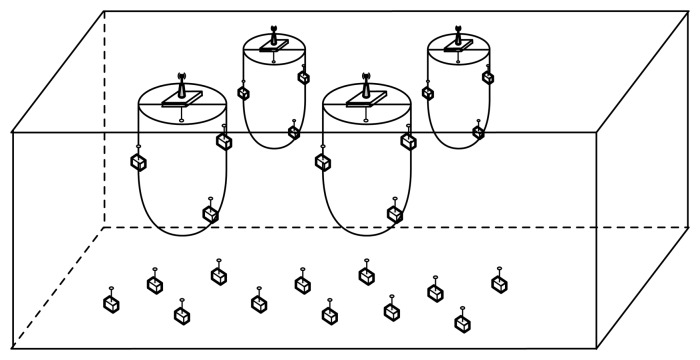
Simulated scenario diagram.

This scenario is part of a Spanish research project involving an unattended monitoring installation in an offshore fish farming facility. The general architecture is depicted in Figure 5, where different sensor nodes are placed near the fish nets and the sea bottom. These nodes are capable of measuring various environmental variables on demand and sending the data to the sink.

Sink nodes (there might be more than one sink node, depending on the installation requirements) are placed on buoys on the sea surface and equipped with solar energy-harvesting capabilities. They also include a radio modem to communicate with an onshore installation.

A sink might require a group of nodes to periodically send some environmental variables. To keep the architecture as flexible as possible, there is no need for these nodes to be known *a priori*. For example, the sink can send a message asking for certain information and the desired sample frequency. The nodes that are capable of providing this information have to compete to acquire the channel and send the information.

Finally, all nodes are equipped with a low-power, low-cost underwater acoustic modem with integrated wake-up capabilities, as presented by Sanchez *et al.* in [8].

### 4.2. Simulation Experiments and Results Using the Energy-Harvesting Module

The energy consumption of one node is studied by varying different parameters and their contribution to system behavior.

To perform this evaluation, we have set a basic scenario:

(i) Network topology is set to a star. Sensor nodes communicate to one single node that acts as the network coordinator and packet sink.
(ii) The network coordinator power-supply is uninterrupted. The rest of the sensor nodes are powered using an energy harvesting system.
(iii) The MAC protocol selected is ALOHA. Although throughput can be severely restricted, channel access time is deterministic. No acknowledgement (ACK)messages are sent in order to minimize network traffic.
(iv) Sensor measurement frequency is configurable in each node.
(v) Nodes can transmit and receive information from the coordinator. For that purpose, sensor nodes will listen to the channel and wait for incoming packets.

**Figure 6.**
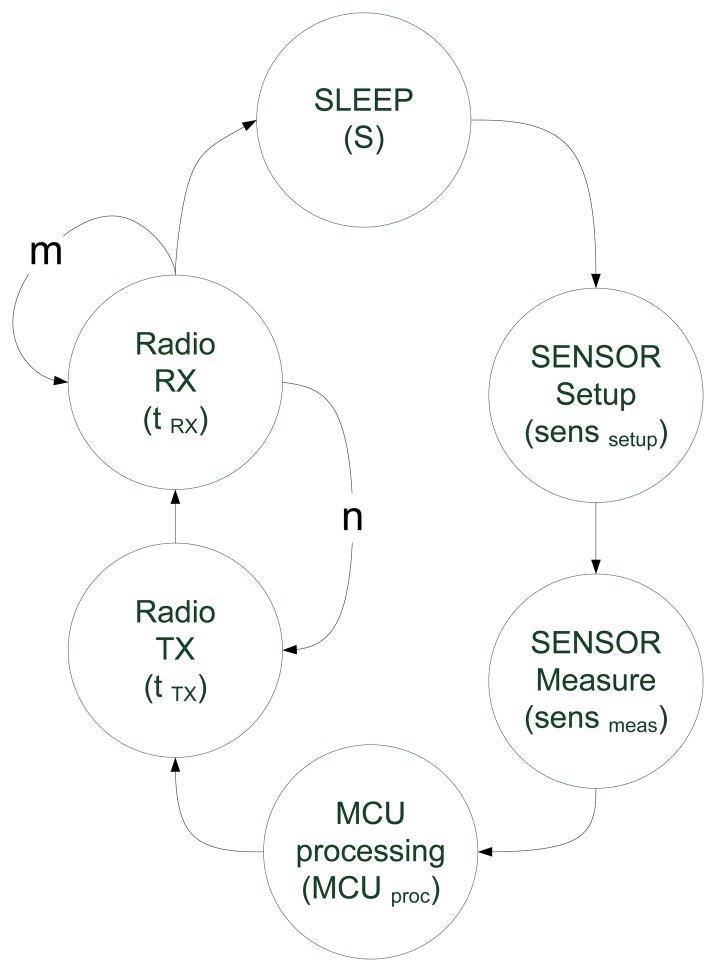
Sensor node simplified state diagram. Remaining time in determined state values are taken from Table 1 and S is the node sleep time (60 s by default).

Basic operation of the sensor node under these circumstances is specified in Figure 6, where n is the number of TX + RX cycles that are performed by the node before going to SLEEP mode again and m is the number of consecutive times the radio remains in receiving state.

As part of the experiments, the energy stored in just one node is analyzed, since the rest present the same behavior. Node current consumption of the various states (Figure 6) is shown in Tables 2 and 3. The duration of each state is given in Table 1.

Capacitor capacitance was set to 50 farads, starting voltage to 0 V and maximum voltage to 2.3 V. Solar radiation data corresponds to the radiation measured by AEMet at Valencia (Spain) on 3 April 2011.

In all scenarios, the simulation stop time was set to 48 hours, and the sensor is only enabled during the sensor-enabled state. During this state, the radio remains in sleep mode.

**Figure 7.**
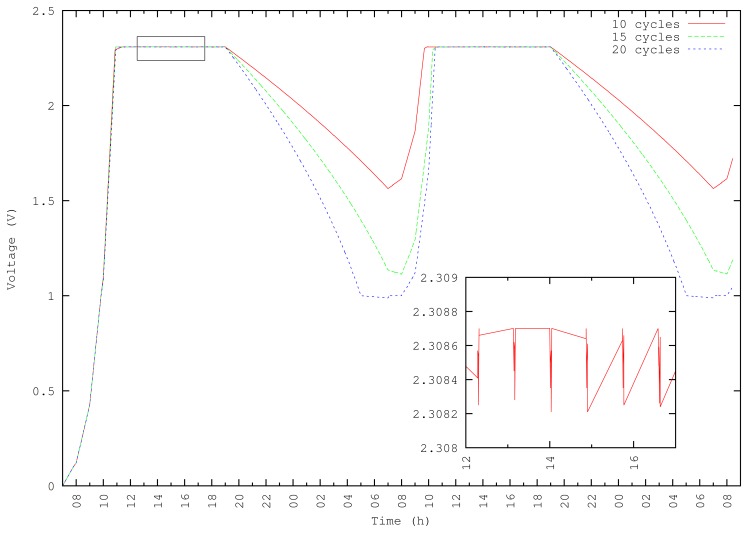
TX/RX cycles variation.

Figure 7 depicts super-capacitor charge and discharge curves during 48 hours. In the node behavior state diagram in Figure 6, the *n* parameter is reconfigured to vary the number of TX + RX loops per period. At sunrise, charge curves match similarly in three cases (n = 10, 15 and 20 loops). The zoom during daylight corresponds to the 10 loop simulation and shows that discharge during daylight is negligible.

Discharge curves start at sunset and differ in each studied case. With 10 and 15 TX/RX loops, the node never stops its operation, since capacitor voltage never falls below 1 V. The energy consumed at night at 20 loops is more than the super-capacitor can provide, and the node stops operation before sunrise.

At sunrise on the second day, the super-capacitor starts receiving energy from the solar panel. During super-capacitor recharge, the node is still transmitting and receiving. This activity can be observed in the figure. However, the node consumes very little energy, and the capacitor can be recharged.

**Figure 8.**
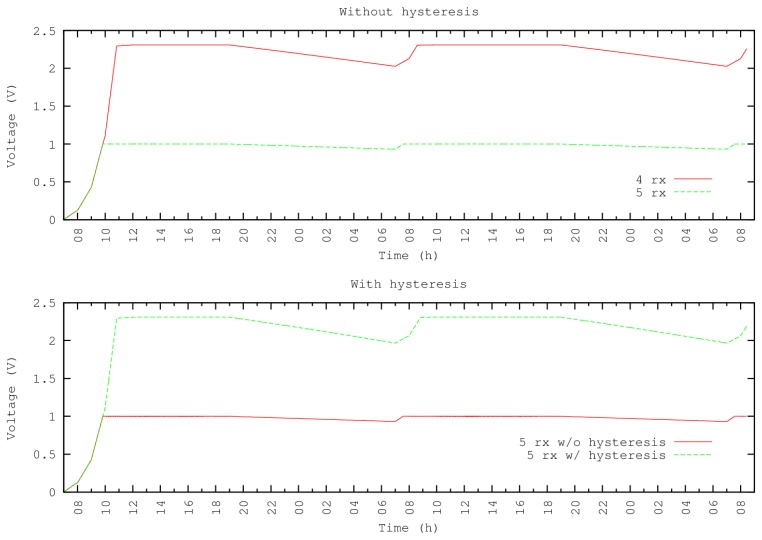
Increasing the RX time by *m* = 4 and *m* = 5 times.

Despite increasing the number of loops, experiments show that nodes receive a quick recharge. Figure 8 shows other experiments where the energy demands at sunrise are greater than the energy provided by the panel, and consequently, the node is unable to recover.

In this experiment, we define the duration of the RX state as *m*×102.5 *ms*, where *m* is a configurable variable for each experiment set as *m* = 4 and *m* = 5. The remaining parameters are as follows: time for one packet transmission = 0.57 ms and time in Sleep mode (S) is 60 seconds. With *m* = 5 nodes, high energy demands prevent it from communicating, since when the capacitor voltage is below 1V, the node remains disabled. When the capacitor voltage reaches the 1 V minimum, it starts an operative cycle: however, the radio RX mode causes the voltage to fall below 1 V again—thereby producing an unrecoverable energy node failure. To solve this issue, we use a comparator with hysteresis to store enough energy to carry out a complete cycle before the node becomes active.

The results of applying this technique are shown in Figure 8. The first part of the figure shows the behavior of the node without hysteresis (when *m* = 5, the node is unable to start operation). The second part of the figure shows that when using hysteresis, the node can start operations and then performs as expected.

### 4.3. Simulation Experiments with MAC Protocols and the Wake-Up Modem

Given the scenario constraints when the underwater nodes have to remain operative and unattended during long periods of time and the network load is not periodically and uniformly distributed, it was decided that ALOHA and MACA-like protocols were more appropriate. These protocols do not need time synchronization and can adapt to the number of contending nodes by using the backoff mechanism. The interested reader can find a more comprehensive study on protocol selection in [12].

The specific protocols chosen for this study are ALOHA-CS, MACA [13], FAMA [14] and T-Lohi [15]. These protocols have been adapted (when possible) to take advantage of the modem wake-up capabilities.

The operation of the sensor nodes is as follows. From the beginning, all nodes remain in a low-power state, and whenever a node needs to send a packet, a wake-up signal is initially sent, and the packet follows immediately afterward.

The Bellhop propagation model was used for the simulations. It was fed with the SSPand bathymetry data, obtained using the WOSS API [16], for the Spanish Mediterranean coast near Burriana, where the application is to be deployed. The modem consumption parameters are shown in Table 4, and the transmission speed was set to 1,000 bps [8].

A scenario of 100 × 100 meters was tested with 10, 50 and 100 nodes randomly deployed. Only one sink was placed at the center of the scenario. Each scenario was simulated several times in order to achieve a confidence interval of ±1 with a confidence level of 95%. All simulations were seeded using the number 1330703057, independent replications were performed advancing the run number [17] and the simulation stop time was set to 30 minutes.

All traffic was directed to the sink node and was generated by a Poisson distribution with an average packet inter-arrival time of 0.6 seconds, which ensured that the network was working under saturation. Each packet was generated by this function and assigned to a source node using a random uniform distribution.

To obtain the best possible results for each protocol regardless of the backoff algorithm used, a set of simulations was performed for each protocol by varying the backoff time from 0.5 seconds to 20 seconds. The best results in terms of packet delivery ratio are shown in the following figures and specified in Table 5. T-Lohi has no backoff time specified, since it is fixed in the protocol specifications [15].

The wake-up modem has two wake-up modes [8,9], a tone or broadcast mode and a selective mode. The tone mode wakes every node that receives the wake-up signal, while the selective mode only wakes the intended receiver. Only the MACA protocols learn, and T-LOHI Tone protocols use the tone or broadcast mode. However, T-LOHI always uses the tone mode during the contention period to decide who is going to be awarded the channel, although the data packet can be sent using either mode.

The results of the simulations follow for each of the implemented protocols using and not using acknowledgments.

#### 4.3.1. Without Acknowledgment

**Figure 9.**
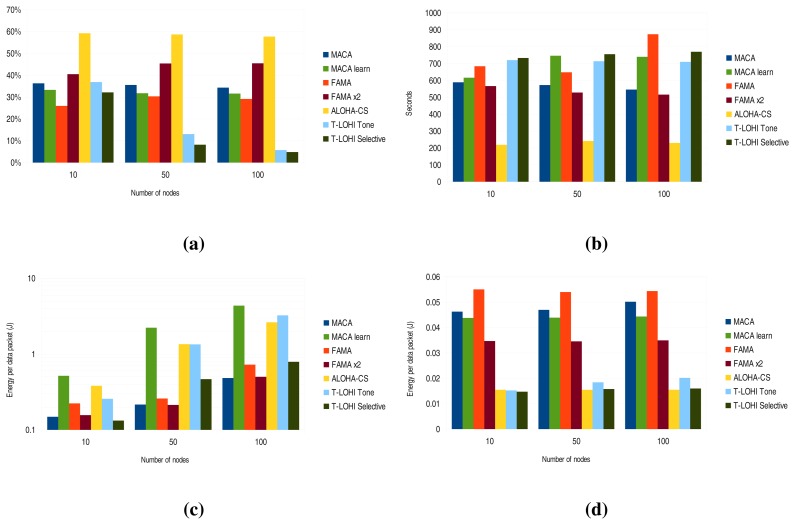
Results without acknowledgment. (**a**) Normalized received packets; (**b**) delay; (**c**) energy per data packet—node; (**d**) energy per data packet—sink.

Figure 9(a) depicts the number of packets correctly received by the sink (normalized to the theoretical maximum). It can be seen that ALOHA-CS achieves the maximum throughput, followed by FAMA x2, which is exactly as productive as FAMA, but sends two data packets instead of one after the RTS/CTS exchange. In this scenario, ALOHA-CS outperforms all the other MAC protocols, because it does not have the control packet overhead, while the collision overhead is small, due to the short packet length. The following section shows that when the transmission speed is reduced by two, packet length doubles and that the FAMA x2 protocol then offers higher throughput.

The MACA learn protocol is exactly the same protocol as the implemented MACA protocol, but uses the wake-up tone capability instead of the selective mode. In this way, all nodes can learn about the on-going transmissions and avoid collisions. As can be seen from the results, we do not achieve better results using this mechanism, due to the higher transmission delay.

Another conclusion extracted from this figure is that the T-LOHI protocol is very density-dependent, and it may be infeasible to use it in high density networks.

Depending on the application requirements, delay could be another important factor for further study. Results in Figure 9(b) show that ALOHA-CS has the lowest delay followed by MACA and FAMA x2. In turn, due to their operation, the two T-LOHI flavors show long delays—with the exception of FAMA when using 100 nodes.

When looking at the power consumption in Figure 9(c), it can be seen that the wake-up tone use of the MACA learn and T-LOHI tone protocols greatly increases the energy consumption of the nodes, since they wake for almost every transmission. It can also be seen that ALOHA-CS has a huge energy consumption per each correctly received packet and that energy consumption overhead reduces when using FAMA x2 instead of FAMA.

Focusing on the sink energy consumption as depicted in Figure 9(d), protocols that send a CTS packet back to the source node have high energy consumptions—the lowest being FAMA x2. Protocols that do not employ CTS packets have the lowest energy consumption.

#### 4.3.2. With Acknowledgment

**Figure 10.**
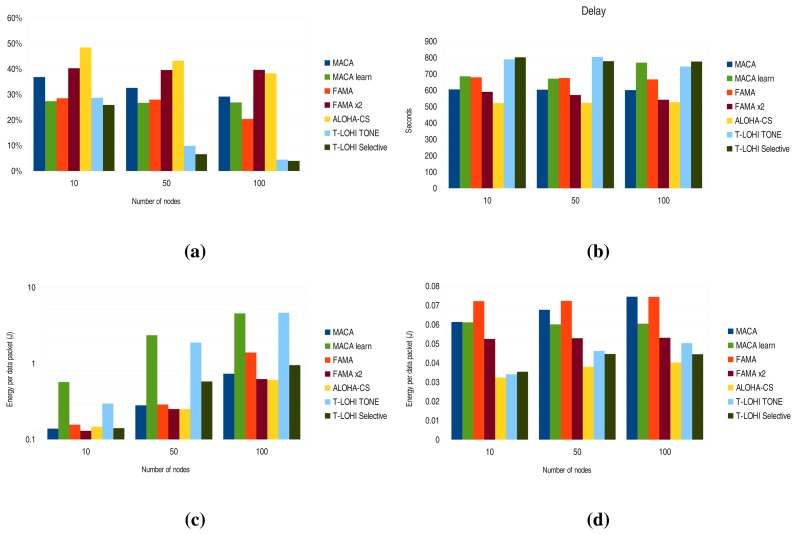
Results without acknowledgment. (**a**) Normalized received packets; (**b**) delay; (**c**) energy per data packet—node; (**d**) Energy per data packet—sink.

When acknowledgments come into play, the number of correctly received packets is reduced, since more time is needed to send the acknowledgments. Also, as shown in Figure 10(a), the difference in the number of correctly received packets is greatly reduced between ALOHA-CS and FAMA x2. Moreover, in the 100-node scenario, FAMA x2 received more packets than ALOHA-CS.

In terms of packet delay, Figure 10(b) shows some interesting results. The delay when using T-LOHI increases slightly, and the larger increment comes with the ALOHA-CS protocol. The remaining protocol delays are fairly stable and do not vary when acknowledgments are introduced.

Figure 10(c) shows how the lowest energy consumption is achieved by FAMA x2 and ALOHA-CS. MACA learning and T-LOHI tone have the highest energy consumptions, as they use the wake-up tone mode.

Finally, sink energy consumption per data packet is depicted in Figure 10(d), and as expected, the protocols without the CTS overhead have the lowest energy consumption. However, since the sink has to transmit and issue acknowledgments, the difference between these protocols and those with CTS is reduced. In addition, it can be seen how FAMA and FAMA x2 have a stable energy consumption, independently of the number of nodes—and this feature may be useful when trying to implement energy-neutral operations at the sink node.

#### 4.3.3. Impact of Transmission Speed

Depending on environmental conditions, nodes may need to lower their transmission speed to decrease the packet error rate. In this section, we are going to analyze how reducing the transmission speed by a factor of two affects the behavior of the protocols when not using acknowledgments.

**Figure 11.**
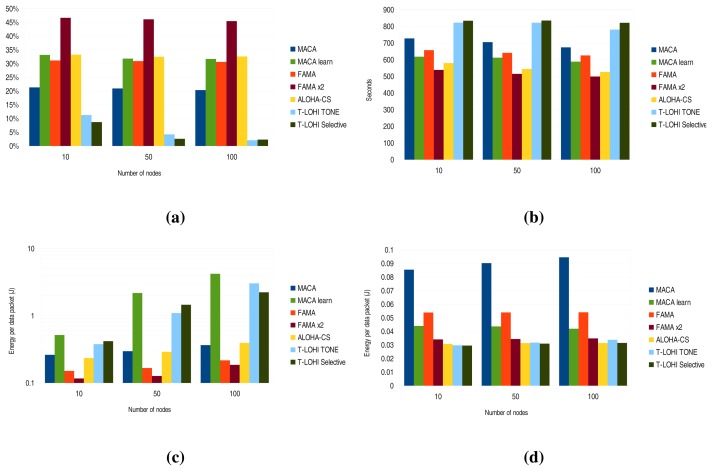
Results without acknowledgment. (**a**) Normalized received packets; (**b**) delay; (**c**) energy per data packet—node; (**d**) energy per data packet—sink.

The first interesting result depicted in Figure 11(a) can be seen in the ALOHA-CS and FAMA x2 protocols. Reducing the transmission speed makes the data frames larger, and this leads to an increase in collisions when using ALOHA-CS. However, the difference between data frames and propagation delay is reduced, since FAMA x2 is much more efficient in channel utilization.

There are also differences in terms of packet delay. Figure 11(b) shows FAMA x2 outperforming the other protocols. However, the delay when using ALOHA-CS increases substantially.

Figure 11(c) shows the energy consumption per correctly received packet for the regular nodes. It can be seen how FAMA and FAMA x2 outperform the other protocols, since the overhead of the large RTS/CTS packets is reduced and collisions are more frequent for the other protocols.

Finally, sink energy consumption per correctly received packet is shown in Figure 11(d). It can be seen that MACA produces the worst results and how the overhead of sending CTS packets in FAMA x2 is minimal when comparing to the energy spent by ALOHA-CS.

## 5. Conclusions

A complete ecosystem of models for the ns-3 simulator has been presented. The utility of the ecosystem has been shown by simulating a scenario that is part of a research project involving the unattended monitoring of an offshore fish farming facility. Moreover, since this platform enables the use of real meteorological data and marine conditions from the location of the installation, the simulations are accurate and provide useful data before deployment.

As future work, new MAC protocols will be implemented, as well as routing protocols to support multi-hop UWSN. Moreover, since network alive time is a major concern, energy-neutral algorithms will be studied by implementing the cooperation of the energy-harvesting model with the wake-up modem and MAC models.

## Figures and Tables

**Table 1.** Sensor states duration time. MCU, microcontroller.

**Task**	**Symbol**	**Time**
Sensor setup	*sens_setup_*	33 ms
Sensor measure	*sens_meas_*	485 *μ*s
MCU data processing	*MCU_proc_*	10.5 ms
Node transmission mode	*t_TX_*	570 *μ*s
Node receive mode	*t_RX_*	102.5 ms

**Table 2.** Radio current consumption.

**MODE**	**I**
TX mode	30 mA
RX mode	27 mA
IDLE	27 mA
SLEEP mode	12 *μ*A

**Table 3.** Sensor current consumption.

**MODE**	**I**
Sensor enabled	6 mA
Sensor disabled	0 mA

**Table 4.** Underwater modem energy consumption.

**MODE**	**Wake-up**	**Modem**
TX mode	120 mW	120 mW
RX mode	8.1 *μW*	24 mW
IDLE mode	8.1 *μW*	24 mW
SLEEP mode	-	3 *μW*

**Table 5.** Backoff time used in the simulations (seconds).

**Number of nodes**	**10**	**50**	**100**
MACA	7.5	26.5	40
MACA Learn	2.5	15	32.5
FAMA	1.5	7	11.5
FAMA x2	2	8.5	15
ALOHA-CS	4	17	32
